# Live-attenuated H1N1 influenza vaccine candidate displays potent efficacy in mice and ferrets

**DOI:** 10.1371/journal.pone.0223784

**Published:** 2019-10-14

**Authors:** Charles B. Stauft, Chen Yang, J. Robert Coleman, David Boltz, Chiahsuan Chin, Anna Kushnir, Steffen Mueller

**Affiliations:** 1 Codagenix, Inc., Farmingdale, New York, United States of America; 2 Life Sciences Group, IIT Research Institute, Chicago, Illinois, United States of America; University of South Dakota, UNITED STATES

## Abstract

Currently, influenza vaccine manufacturers need to produce 1–5 x 10^7^ PFU of each vaccine strain to fill one dose of the current live-attenuated-influenza-vaccine (LAIV). To make a single dose of inactivated vaccine (15 ug of each hemagglutinin), the equivalent of 10^10^ PFU of each vaccine strains need to be grown. This high dose requirement is a major drawback for manufacturing as well as rapidly sourcing sufficient doses during a pandemic. Using our computer-aided vaccine platform *S*ynthetic *A*ttenuated *V*irus *E*ngineering (*SAVE*), we created a vaccine candidate against pandemic H1N1 A/CA/07/2009 (CodaVax-H1N1) with robust efficacy in mice and ferrets, and is protective at a much lower dose than the current LAIV. CodaVax-H1N1 is currently in Phase I/II clinical trials. The hemagglutinin (HA) and neuraminidase (NA) gene segments of A/California/07/2009 (H1N1) (CA07) were “de-optimized” and a LAIV was generated *ex silico* using DNA synthesis. In DBA/2 mice, vaccination at a very low dose (10^0^ or approximately 1 PFU) with CodaVax-H1N1 prevented disease after lethal challenge with wild-type H1N1. In BALB/c mice, as little as 10^3^ PFU was protective against lethal challenge with mouse-adapted H1N1. In ferrets, CodaVax-H1N1 was more potent compared to currently licensed LAIV and still effective at a low dose of 10^3^ PFU at preventing replication of challenge virus.

## Introduction

The recent 2017–18 flu season in the US was as severe as the 2009 pandemic and contributed to at least 133 pediatric deaths [1]. This led to an invigorated response from the US government–with NIAID releasing a plan on investing in new flu vaccines [2] as well as testimony by the CDC, FDA, and BARDA to the US Congress on the need for cell-based vaccines that can be manufactured efficiently and that induce a broad immune response [3].

Influenza virus vaccine development is challenged by the constant adaptations made by the virus to the selective pressure of host immunity. This biological arms race has favored the many subtypes of influenza virus, represented by the phenomena of antigenic drift and shift that underlie the virus’s ability to evade annual attempts to produce effective vaccines. The recent 2017–18 influenza season in the United States was particularly severe and highlighted the need for cell-based vaccines that can be manufactured rapidly and efficiently, and that induce a broad immune response.

The generation of live attenuated vaccines has long been hampered by the lack of a rational approach to their design. All currently licensed, live, viral vaccines have been developed essentially by trial and error, by extensive serial passaging of viruses through non-human cells or other restrictive conditions, hoping the resulting virus will be sufficiently attenuated. The attenuation phenotype of such traditional live vaccines is generally not understood and is often based on very few amino acid changes, making them liable to potential reversion to a more virulent form. Furthermore, the serial passaging process and the ensuing mutations can alter the immunogenic properties of the resulting vaccine strain.

We have developed an innovative technology called “Synthetic Attenuated Virus Engineering” (*SAVE*), as a rational approach to building live-attenuated viruses[4]. *SAVE* is based on recoding a portion(s) of a target viral genome without altering the amino acid sequence of the wild-type virus, a process termed human-cell “deoptimization.” Deoptimization is achieved by using a computer algorithm to rearrange existing synonymous codons to create a sub-optimal arrangement of pairs of codons (codon pair bias design)[4,5]. The *SAVE* approach capitalizes on the fact that the genetic code is redundant and flexible, and that all viral pathogens must translate their genome into proteins using the host cell's translational machinery. In the human host, multiple codons are used at varying frequencies in a gene to encode the same amino acid, with some codons being more or less “rare” compared to what would be predicted (i.e. codon bias), likewise, pairs of codons can be under-represented or over-represented in a given nucleotide sequence where adjacent codons for a given amino acid pair occur at varied/unexpected frequencies [i.e. codon pair bias (CPB)]. By scrambling existing synonymous codons within a sequence to increase the frequency of sub-optimal codon pairs based on a host codon pair bias matrix, *SAVE*-deoptimization introduces many hundreds or thousands of nucleotide changes which act in a synergistic manner to introduce attenuation and make reversion to virulence unlikely, while the encoded protein sequence remains unchanged [6]. Mechanisms of attenuation by altering CPB are still being elucidated but commonly involve reduction of protein synthesis in human influenza virus [7,8].

*SAVE* is a platform that targets the fundamental process of translation and has been shown to be effective, yielding candidate live vaccines for poliovirus [4], respiratory syncytial virus [9,10], dengue 2 virus [11], and influenza A virus [7,8,12], amongst others. *SAVE* deoptimization results in a candidate that is, at the protein level, identical to the wild-type virulent strain and this exact antigenic match allows for high immunogenicity at low doses[8]. The 2009 pandemic and resurgence of influenza in the 2017–2018 season served as wake up call for the need for more effective influenza vaccines that can be sourced rapidly[13]. Here we describe applying *SAVE* to the pandemic H1N1 A/CA/07/2009 –seeking to demonstrate this ultra-low dose capability in a clinically relevant strain of influenza A virus.

The two types of influenza vaccines available commercially are the cold-adapted live attenuated vaccine (LAIV) and influenza inactivated vaccines (either trivalent or quadrivalent, IIVs). Both vaccine types have significant drawbacks. IIVs are intramuscularly administered and require approximately the equivalent of 10^10^ plaque-forming units (PFU) worth of hemagglutinin (HA) antigen per dose. This is equivalent to one egg per shot which has led to vaccine shortages in the past [14]. The ever-present danger of an egg shortage either from vaccine demand or from avian influenza reducing egg production highlights the need for alternative approaches to manufacturing highly effective and safe influenza vaccines [15]. Additionally, IIVs are inert, antigen based vaccines, unable to induce significant cell-mediated immunity [16], and hence demonstrate low efficacy (as low as 9%) in at risk populations like the elderly [17].

LAIVs are capable of inducing cellular in addition to humoral immunity but the current, licensed LAIV, FluMist® trivalent and quadrivalent, is restricted to use by people 2–49 years of age in the United States and has shown tolerance/low efficacy in repeat recipients [8]. The FluMist® technology is based on a backbone of cold-adapted genes from a strain that is nearly 60 years old, combined with the major antigenic markers HA and neuraminidase (NA) from annual, recommended vaccine strains [18]. The low efficacy and a high dose requirement of both IIVs and FluMist® dictate the need for improved influenza vaccines.

CodaVax-H1N1 was designed by recoding the HA and NA segments of the wild-type pandemic H1N1 strain A/California/07/2009 (CA07). The mutations were introduced using a previously developed algorithm[5] of rearranging synonymous codons within each segment, to create a plurality of sub-optimal codon pairs, without changing codon usage or amino acid sequence. This process resulted in virus attenuation while preserving the immunogenicity of wild-type virus [7,12]. In a study conducted by NIH-NIAID, protein expression of these segments was reduced by approximately 40–50% as compared to *wt* CA/07/09 [7]. The segment-specific reduction in protein expression leads to a powerful vaccine candidate, as CodaVax-H1N1 is attenuated yet still expresses wild-type antigenic targets to the host immune system.

## Results

CodaVax-H1N1 was designed by recoding the segments of the pandemic H1N1 strain CA07 encoding the HA protein (346 synonymous mutations; 24% nucleotide difference to wt) and NA protein (293 synonymous mutations; 24% nucleotide difference to wt). The mutations were introduced by rearranging the codons in the wt genes to form suboptimal pairs of codons for both segments without changing codon usage, resulting in a high degree of attenuation. In MDCK cell culture, replication kinetics of CodaVax-H1N1 [7,8] are preserved despite attenuation *in vivo* [7]. This is promising as reduced growth *in vitro* would diminish the ability of the virus to be grown for vaccine production. Additionally, CodaVax-H1N1 is not temperature-sensitive as growth kinetics were not reduced in MDCK cells at 39°C, demonstrating that the *SAVE* redesigned CodaVax-H1N1 can be grown to high titers with cell-based production methods. CodaVax-H1N1 has already been shown to be highly attenuated, immunogenic, and protective in swine [12]. In this study, we sought to demonstrate the low-dose potential of CodaVax-H1N1, protection in mouse and ferret models, and to compare these characteristics with a currently licensed LAIV.

### Attenuation, immunogenicity, and protection from lethal challenge in CodaVax-H1N1 vaccinated mice

**Fig 1:**
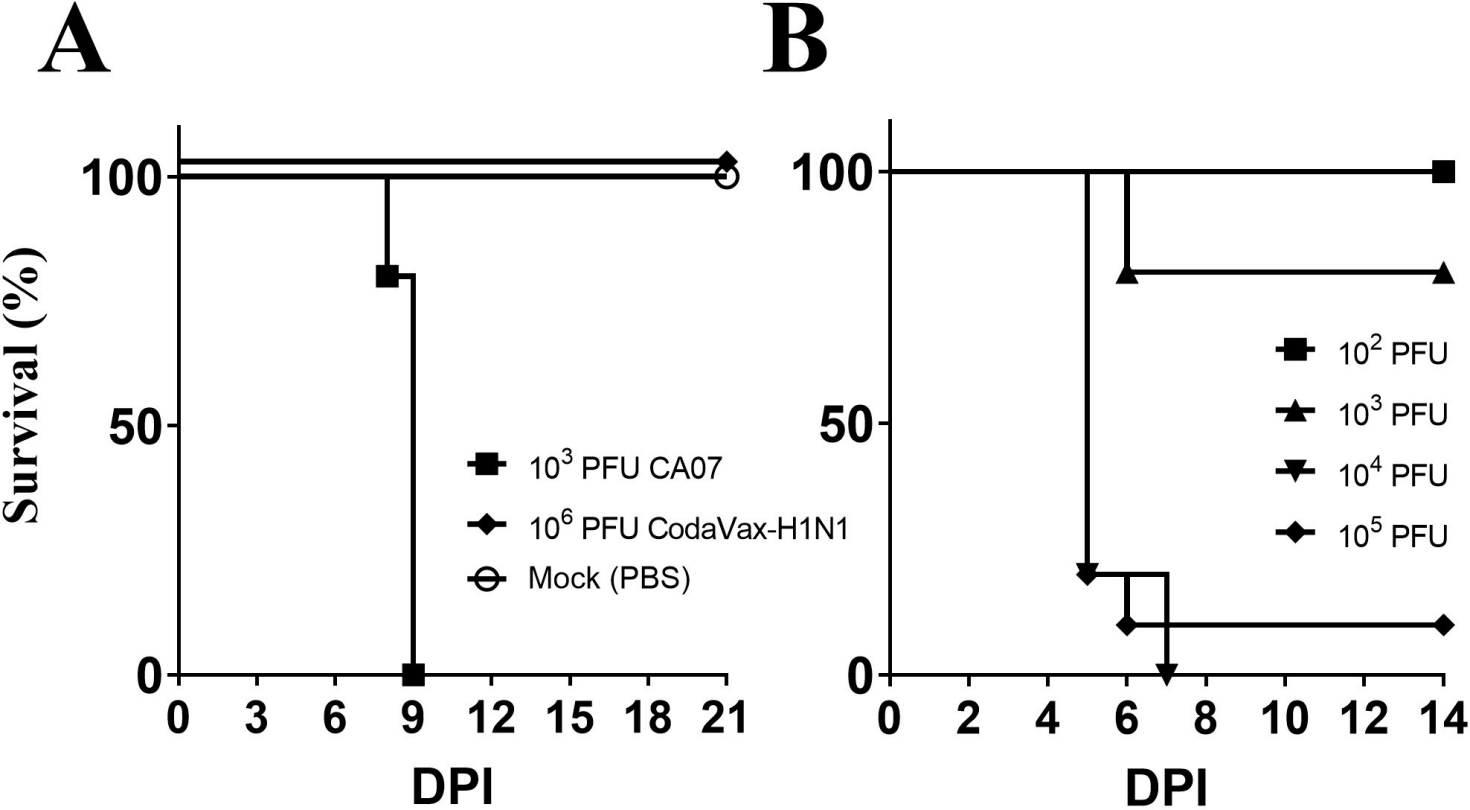
DBA/2 and BALB/c challenge models for H1N1. Wild-type CA07 (10^2^, 10^3^ PFU) and CodaVax-H1N1 (10^6^ PFU) were used to infect DBA/2 (A) and CA07ma used to infect BALB/c (B) mice by the intranasal route in a volume of 25 μl. In DBA/2 mice, the LD_50_ of wild-type CA07 was 3.2 x 10^2^ PFU while the LD_50_ of CodaVax-H1N1 was ≥3.2 x 10^6^ PFU, at least a 10,000-fold difference. CA07ma had an LD_50_ of 4.2 x 10^2^ PFU while neither CA07 WT nor CodaVax-H1N1 caused morbidity or mortality in BALB/c mice. DPI: Days Post Infection.

We used DBA/2 mice to assess virulence and vaccine efficacy because BALB/c mice do not show severe weight loss when infected with wild-type CA07 virus. DBA/2 mice have been used previously as a sensitive model of influenza virulence for pandemic H1N1 [19] as well as strains of H5N1 [20]. DBA/2 mice were infected via the intranasal route with 25 μL of ten-fold diluted concentrations of wild-type virus and CodaVax-H1N1 viruses to assess virulence by Kaplan-Meier kinetics and LD_50_ determination. CodaVax-H1N1 virus demonstrated >10,000-fold attenuation in DBA/2 mice compared to wild-type virus (Fig 1). Wild-type virus had an LD_50_ of ~3.2 x 10^2^ PFU while CodaVax-H1N1 virus did not cause any mortality at a dose of 1 x 10^6^ PFU (Fig 1A), or a theoretical “worst case” LD50 of ≥3.2 x 10^6^.

As a lethal challenge model to test vaccination in BALB/c mice, we generated a mouse-adapted CA07 virus (CA07ma) from wt CA07 by introducing two amino acid mutations found in the mouse-adapted A/California/04/2009 (CA04ma) [21]. As predicted, the mouse adaptation mutations behaved similarly in CA07ma as reported for CA04ma. The two mutations increased the virulence of CA07ma compared to wt CA07 by 10,000-fold or more, reducing the LD_50_ in BALB/c mice from > 3.2 x 10^6^ to 5.6 x 10^2^ PFU (Fig 1B). Thus, CA07ma could be used as a challenge virus in vaccinated BALB/c mice in place of the less virulent wild-type.

**Fig 2:**
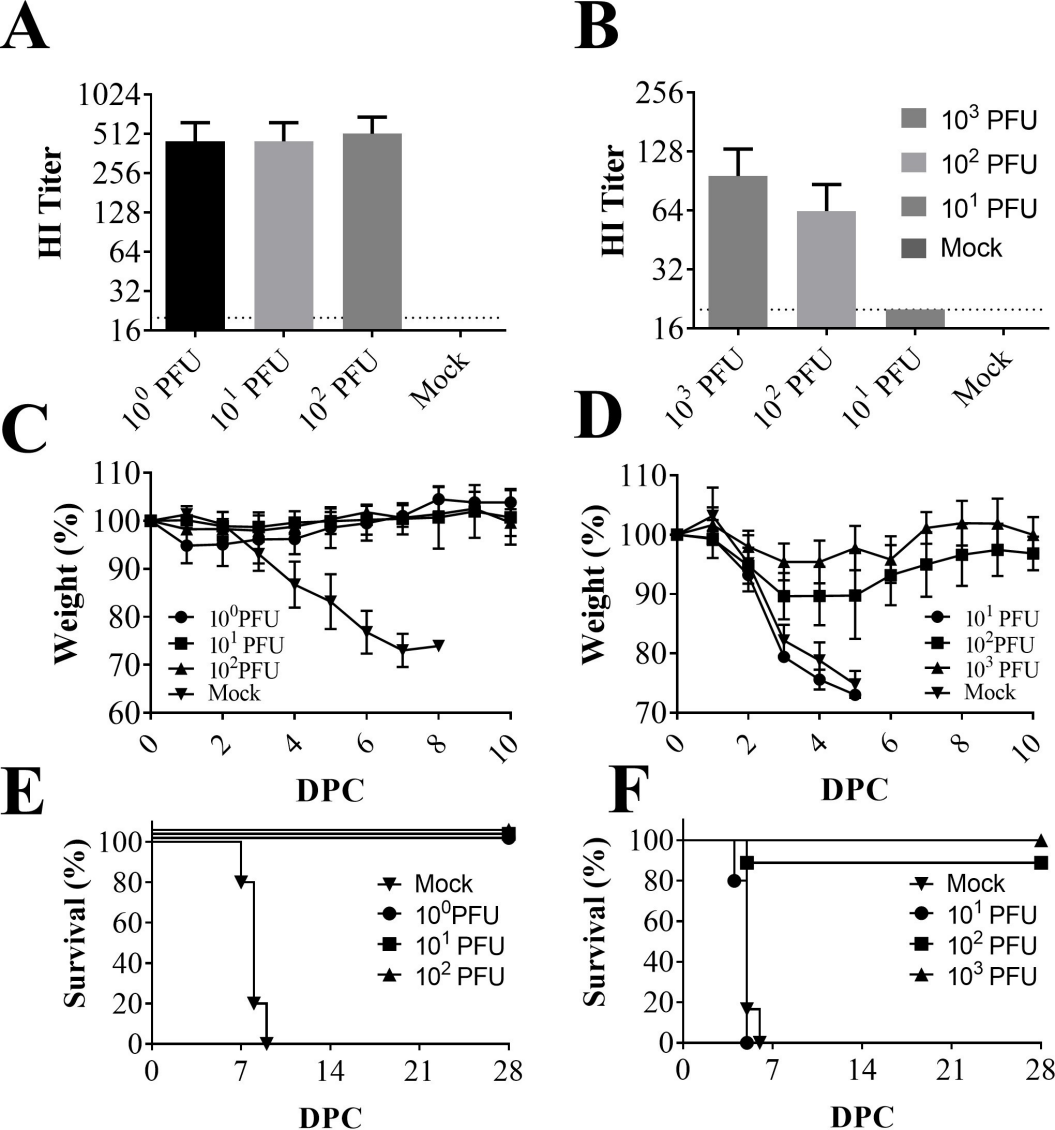
Immunogenicity and protection against lethal challenge in DBA/2 and BALB/c mice models. DBA/2 mice (n = 5) were vaccinated with 10^0^, 10^1^, or 10^2^ PFU CodaVax-H1N1 and BALB/c mice (n = 5) with 10^1^, 10^2^, 10^3^ PFU of CodaVax-H1N1 delivered in 25 μl by the intranasal route. Sera collected at 28 DPI from vaccinated and mock-vaccinated DBA/2 (A) and BALB/c (B) mice were tested for anti-CA07 antibodies using a hemagglutinin-inhibition (HI) assay. High levels of anti-CA07 antibodies were detected in DBA/2 sera at all vaccinating doses, while in BALB/c mice the titers were above mock-vaccinated levels after vaccination with 10^3^ (p = 0.007, Student’s t-test) or 10^2^ (p = 0.002, Student’s t-test) PFU. Vaccination with 10^1^ PFU CodaVax-H1N1 resulted in a ~2-fold seroresponse in all BALB/c mice. Limit of detection (dotted line) was 20, values <20 were set at 10 for analysis. At 28 DPI, DBA/2 mice were challenged with 1 x 10^5^ PFU CA07, with weight loss (C) and mortality (E) used to assess efficacy. BALB/c mice were challenged with 1 x 10^5^ PFU CA07ma at 28 DPI with weight loss (D) and mortality (F) used to assess efficacy. Mortality was determined by the humane early-endpoint of ≥20%. DPC: Days Post-Challenge.

DBA/2 mice (Fig 2A, 2C and 2E) were vaccinated with 10^0^, 10^1^, or 10^2^ PFU CodaVax-H1N1. Immunogenicity was measured by hemagglutination-inhibition (HI) assay of sera collected on 28 dpi. High levels of anti-CA07 antibodies were detected in DBA/2 sera at all vaccinating doses with no significant difference observable between the 10^0^ (GMT 422.2), 10^1^ (GMT 422.2), and 10^2^ PFU (GMT 485.0) vaccinated groups (Fig 2A). Vaccinated and non-vaccinated DBA/2 mice were challenged 28 days post vaccination with 1 x 10^5^ PFU (500 LD_50_) of wild-type CA07 virus. Mice were monitored daily for morbidity (measured by weight loss) and mortality post-challenge to determine protective efficacy. The protective dose 50% (PD_50_) was determined to be very low, approximately 5 PFU. As the LD_50_ in DBA/2 mice was found to be in excess of 10^6^ PFU, this represents a margin of safety [8] of almost 10^6^, with the protective dose being approximately one million times lower than the lethal dose. The lowest dose attempted (1 PFU) protected 100% of mice (n = 5) against mortality post-challenge (Fig 2C and 2E). Unvaccinated mice all lost ≥20% of their initial body-weight by day 11 post-infection while 10^1^ and 10^2^ PFU vaccinated animals did not show appreciable weight loss (Fig 2C).

BALB/c mice were vaccinated with 10^1^, 10^2^, or 10^3^ PFU of CodaVax-H1N1 (Fig 2B, 2D and 2F) and sera were tested for anti-H1N1 antibodies by HI assay at 28 dpi (Fig 2B). The HI titers were above mock-vaccinated levels after vaccination with 10^3^ (p = 0.007, Student’s t-test) or 10^2^ (p = 0.002, Student’s t-test) PFU CodaVax-H1N1. Vaccination with 10^1^ PFU CodaVax-H1N1 resulted in a ~2-fold sero-response in all BALB/c mice, however the HI titers were low (GMT 20.0) compared to the 10^2^ PFU (GMT 63.6) and 10^3^ PFU (GMT 96.0) groups. Although some mice experienced transient weight loss (Fig 2D), mice in the 10^3^ PFU group were completely protected and mice in the 10^2^ PFU group were 89% protected from mortality after challenge with 500 LD_50_ CA07ma, a protective-dose 50% (PD_50_) of 3.2 x 10^1^ PFU (Fig 2F).

### Safety, immunogenicity, and efficacy of CodaVax-H1N1 vaccination in ferrets

To better evaluate CodaVax-H1N1 as a LAIV candidate, we compared the performance of CodaVax-H1N1 with FluMist®, the current, cold-adapted LAIV quadrivalent vaccine using a standard ferret model. We used this model to assess safety, immunogenicity, and protection against challenge with wt CA07.

**Fig 3:**
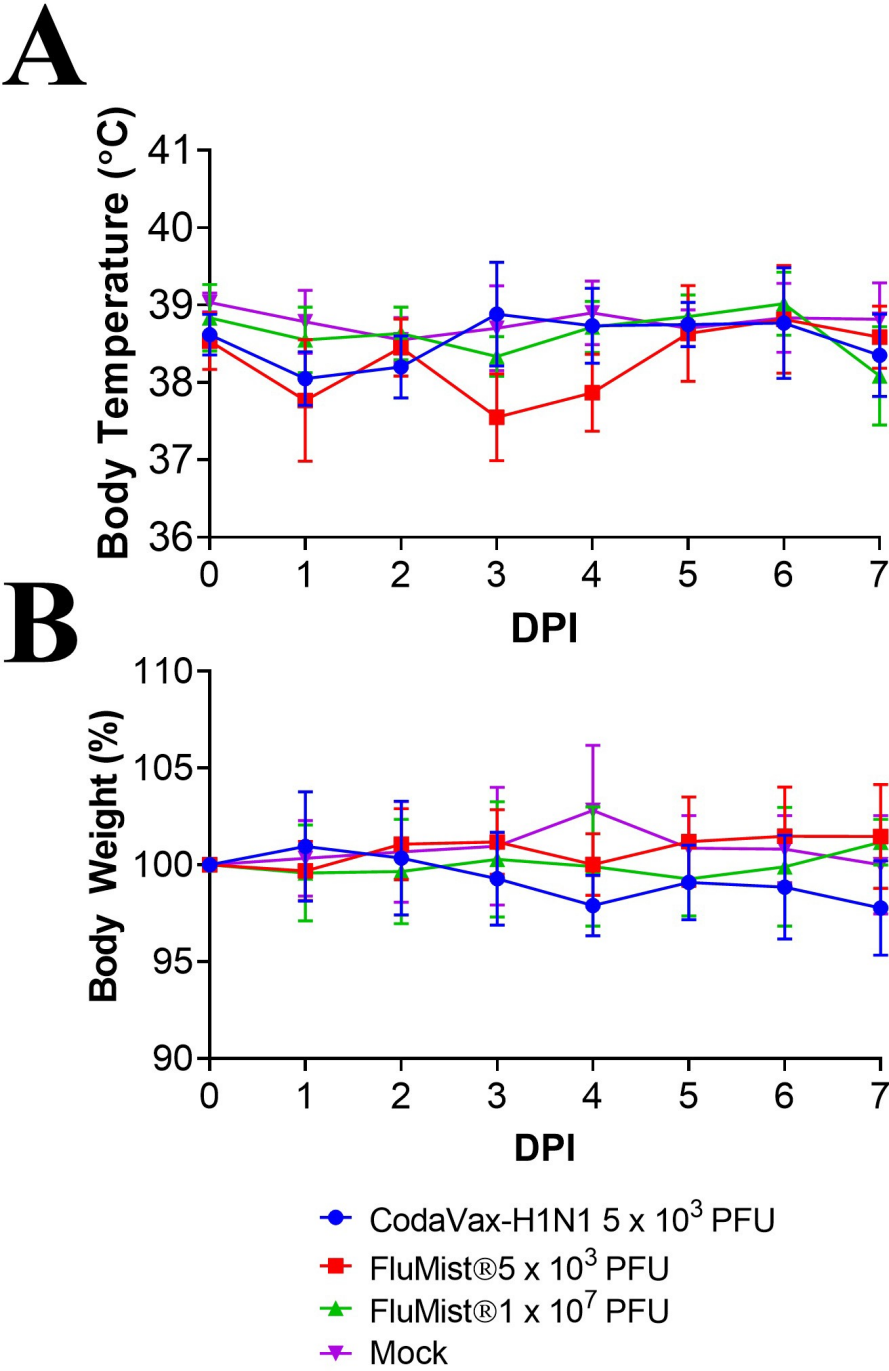
Attenuation in vaccinated ferrets. Ferrets (n = 6) were vaccinated with 5 x 10^3^ PFU CodaVax-H1N1, 5 x 10^3^ PFU FluMist®, 1 x 10^7^ PFU FluMist®, or else mock-vaccinated with vehicle control. Body temperatures and weight were measured for 7 days post-vaccination to assess attenuation. No significant changes in temperature (A) or weight (B) were observed for CodaVax-H1N1 vaccinated ferrets compared to mock controls, similar to FluMist® vaccinated groups.

The safety of CodaVax-H1N1 at a single dose (5 x 10^3^ PFU) and FluMist® at two different doses (5 x 10^3^ or 1:2000 of a commercial human dose, and 1 x 10^7^ PFU, or one full commercial human dose) was compared in ferrets by measuring body weight post challenge. No significant changes in body weight were observed after vaccination with CodaVax-H1N1, comparable to ferrets vaccinated with FluMist®, which also did not exhibit significant changes in body weight (Fig 3).

To compare immunogenicity to the commercial LAIV, four groups of six ferrets each were vaccinated with a single dose of either CodaVax-H1N1 (5 x 10^3^ PFU), FluMist® (5 x 10^3^ or 1 x 10^7^ PFU), or saline. Vaccine-induced anti-hemagglutinin antibodies were determined in serum collected 28 days after vaccination by hemagglutination inhibition (HI) assay against A/California/07/2009 (Table 1).

**Table 1:** HI antibody titers (GMT) in ferrets on days 28 (post-vaccination) and 49 (post-challenge).

Group#	Vaccine	Day 28 (95% CI)	Day 49 (95% CI)
1	5 x 10^3^ PFU CodaVax-H1N1	718.4 (533.8–966.8)	1015.9 (139.1–7419)
2	5 x 10^3^ PFU FluMist®	7.1 (2.9–17.2)	≥5120 (N/A)
3	1 x 10^7^ PFU FluMist®	14.1 (6.6–30.3)	≥5120 (N/A)
4	Mock	5 (5–5)	2560 (2560–2560)

# Four groups of 6 ferrets were vaccinated once on day 0 with the indicated vaccines, and challenged on day 35 with 10^6^ TCID_50_ wild type A/California/07/2009. Serum antibody titers after vaccination (Day 28; N = 6) and 14 days after challenge (Day 49; N = 3) against homologous A/California/07/2009 virus were determined by HI assay.

Of the group receiving the 5 x 10^3^ dose of FluMist®, only one ferret seroconverted (HI titer of 40). Low HI titers, ranging from 10 to 40, were detected in 83.33% of ferrets receiving the full commercial dose of ~1 x 10^7^ dose of FluMist®. Ferrets vaccinated with CodaVax-H1N1 all seroconverted and developed the highest level of post vaccination anti-HA antibodies with HI titers ranging from 640 to 1280 (Table 1). Serum HI titers remained low (≤10) in all of the mock vaccinated ferrets. Serum from CodaVax-H1N1 vaccinated ferrets had significantly higher titers than FluMist® at either 5 x 10^3^ or at the commercial human dose of approximately 1 x 10^7^ PFU (p = <0.001 by Student’s t-test).

**Fig 4:**
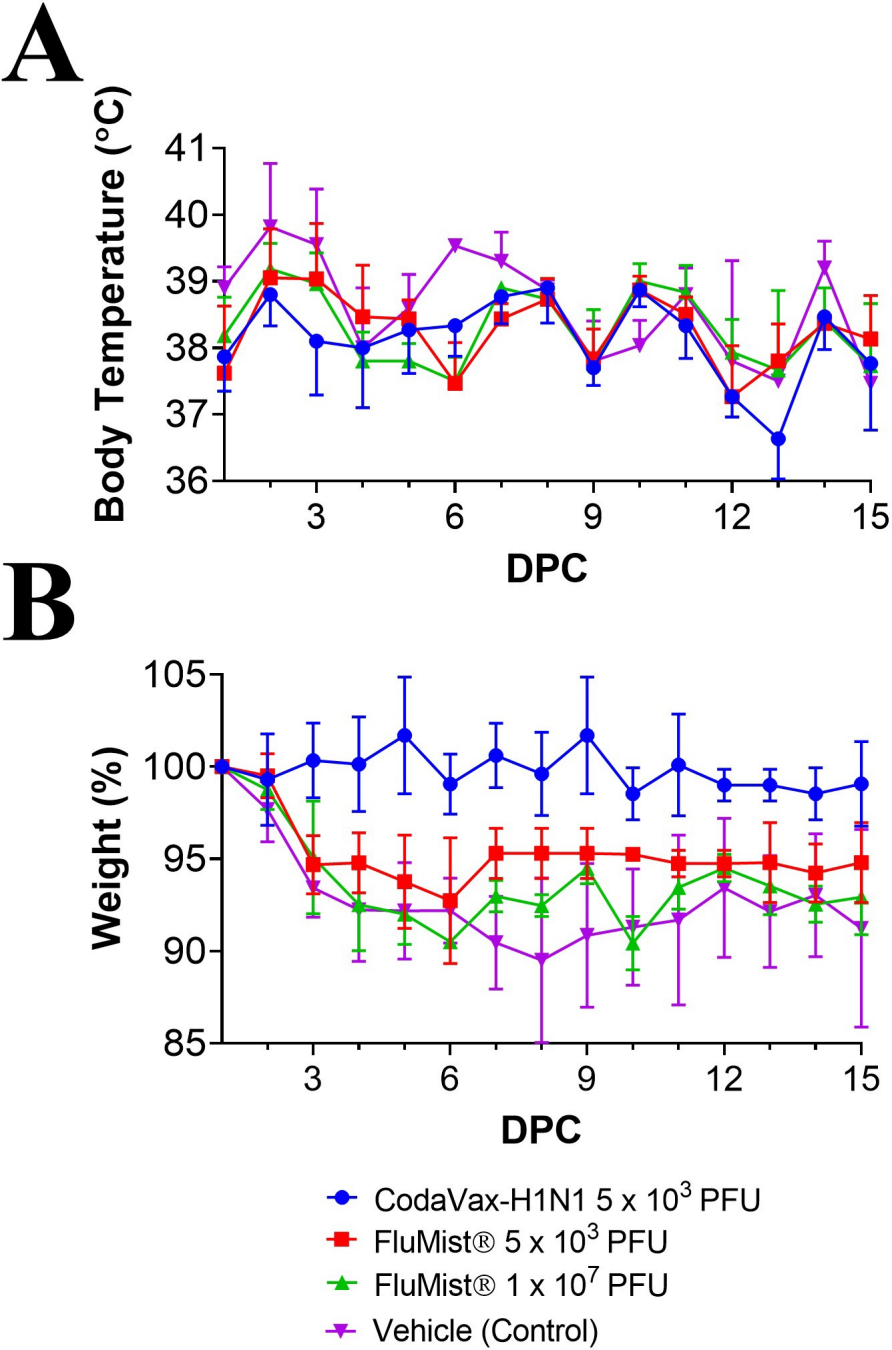
Prevention of morbidity in vaccinated ferrets post-challenge. Ferrets were vaccinated with 5 x 10^3^ PFU CodaVax-H1N1, 5 x 10^3^ PFU FluMist®, 1 x 10^7^ PFU FluMist®, or mock-vaccinated with vehicle control were challenged with 1x10^6^ TCID_50_ of CA07 WT on day 35. Vaccine efficacy was determined using body temperature (A) and weight (B) to measure morbidity for 15 days post-challenge (DPC). Body temperatures in CodaVax-H1N1 vaccinated ferrets were significantly lower than mock-vaccinated ferrets at 1, 2, and 3 days post-challenge while no significant difference in body temperature was observed on days 2–5 for FluMist® vaccinated ferrets. Mean body weight was also higher in CodaVax-H1N1 and 1 x 10^7^ PFU FluMist® vaccinated ferrets compared to mock every day from day 3–15 while the 5 x 10^3^ PFU FluMist® vaccinated group showed protection only at 6, 7 DPC (2-Way ANOVA).

The protective efficacy of CodaVax-H1N1 and the FluMist® quadrivalent vaccination was assessed by challenging all ferrets intranasally with 1 x 10^6^ TCID_50_ of wt CA07 on day 35 after vaccination. A maximum average weight loss of 7.3 to 10.5% was observed between days 5–7 post challenge for vehicle control and FluMist® vaccinated groups (Fig 4). Ferrets vaccinated with CodaVax-H1N1 demonstrated a maximum weight loss of 1.5% observed on day 9. Elevated body temperatures post challenge (Fig 4) were observed on day 1 in all groups. Body temperatures in CodaVax-H1N1 vaccinated ferrets were, however, significantly lower than mock-vaccinated ferrets at several time points (p = 0.002, 1 DPC; p = 0.041, 2 DPC; p = 0.012, 3 DPC; Student’s t-test) post-challenge. Ferrets vaccinated with the human dose (1 x 10^7^ PFU) of FluMist® had reduced fever on day 1 post-challenge (p = 0.024; Student’s t-test), but there was no significant difference in body temperatures between mock and FluMist® vaccinated ferrets on days 2–5 post-challenge (Fig 4).

**Fig 5:**
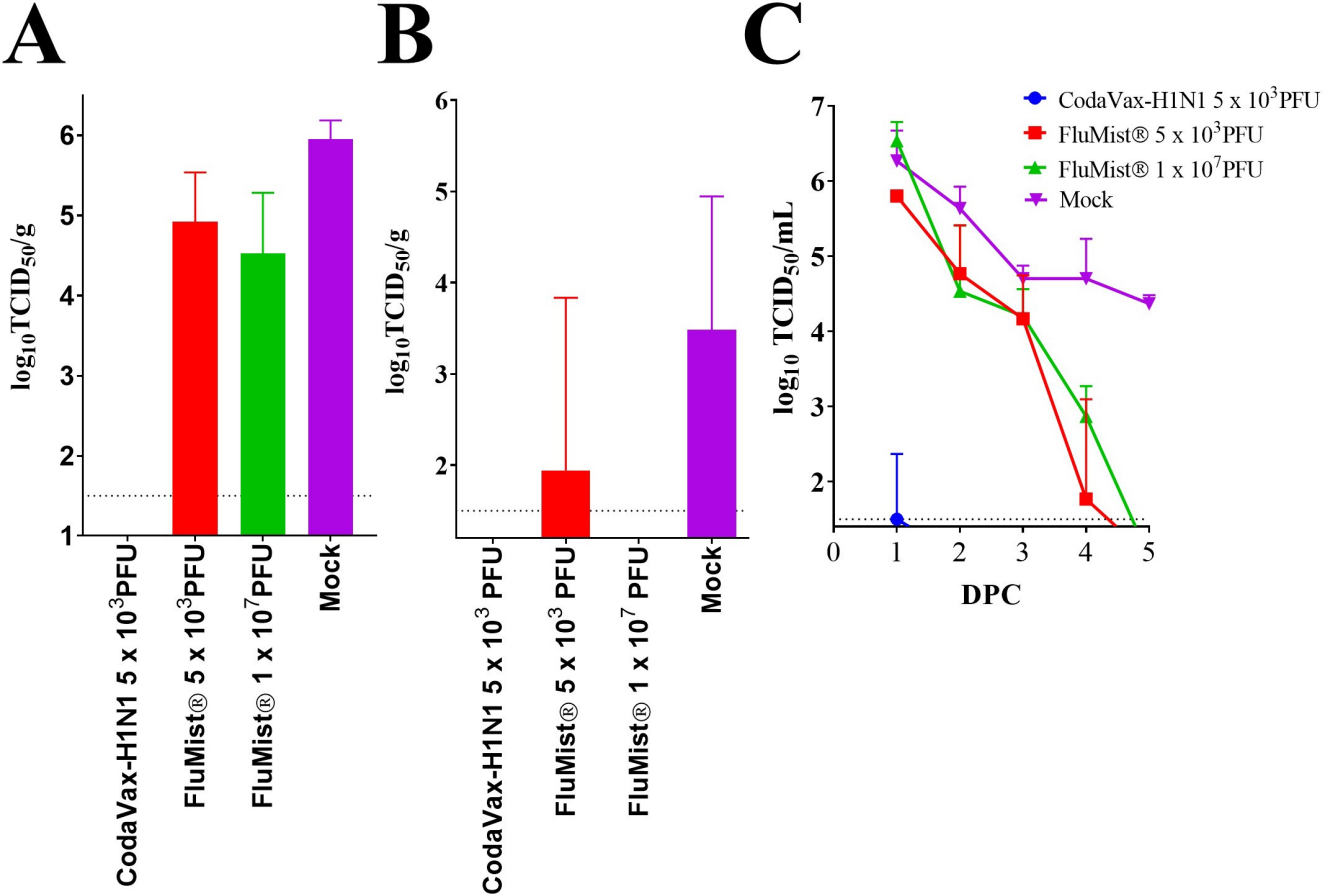
CodaVax-H1N1 shows superior levels of efficacy at a low dose in ferrets compared to the current LAIV comparator Flumist®. Ferrets (n = 6) were vaccinated once with a single dose as indicated followed by challenge with 10^6^ TCID50 of wt A/CA07/2002 (H1N1). Two days after challenge (peak of viral replication) selected animals (n = 3) were euthanized to determine challenge virus loads in nasal turbinate (A) and lung (B) tissues. Nasal wash titers (C) were measured on days 0–5 post-challenge in the remaining 3 ferrets of each group. Dotted line indicates limit of detection. Nasal turbinate titers were not significantly reduced in FluMist® vaccinated ferrets at the highest dose of 1 x 10^7^ PFU (p = 0063; Student’s unpaired t-test) compared to mock-vaccination. Undetectable titers are set at the limit of detection for the assay (1.5 log_10_). DPC: Days post-challenge.

Examination of body weight and body temperature indicated minimal morbidity post-challenge in CodaVax-H1N1 vaccinated ferrets and was confirmed by titration of wt CA07 virus in respiratory samples collected post-challenge. Nasal turbinates (Fig 5A) and lung tissues (Fig 5B) were harvested on day 2 post challenge from three ferrets of each group. Nasal washes (Fig 5C) were collected daily on days 0–5 post challenge to test for virus shedding from the remaining three animals.

Vaccination with FluMist® Quadrivalent at either dose only modestly reduced challenge virus shedding in nasal turbinates as compared to mock vaccinated ferrets (Fig 5A). Of the FluMist® vaccinated ferrets, 2/3 (5 x 10^3^ PFU dose) and 1/3 (1 x 10^7^ PFU dose) ferrets still had detectable virus in one lobe of their lung. However, no detectable virus was found in the lungs of CodaVax-H1N1 vaccinated ferrets. All mock-vaccinated ferrets had detectable virus replication in their lung samples.

Virus shedding in nasal washes of challenged ferrets largely mirrored those observed in nasal turbinates. On days 1 to 3 post challenge, high levels of challenge virus were detected in the nasal washes of all ferrets in both FluMist® vaccinated groups, and in mock vaccinated animals (Fig 5C). Virus titers were similar in the FluMist® vaccine groups compared to the mock vaccinated group. On day 4, only a single 5 x 10^3^ FluMist® vaccinated ferret was shedding virus; whereas, 3/3 ferrets in the 1 x 10^7^ FluMist® group were shedding virus. Only mock-vaccinated ferrets were shedding on day 5. In contrast, none of the CodaVax-H1N1 vaccinated ferrets shed any detectable challenge virus between days 2 and 5 post-challenge, and only one animal did so on day 1, at a greatly reduced level (Fig 5C). Using a Bonferroni adjusted repeated measures one-way ANOVA, the FluMist® nasal wash data were not found to be significantly different from vehicle control (mock). However, CodaVax-H1N1 vaccination was effective in reducing nasal wash titers as titers from CodaVax-H1N1 vaccinated ferrets were found to be significantly lower (p = <0.01 5 x 10^3^ FluMist®; p = <0.01 1 x 10^7^ FluMist®; p<0.001 mock). For this statistical test, undetectable titers were conservatively set at the limit of detection (1.5 TCID_50_/mL).

Finally, sera collected from the ferrets remaining at day 49 (14 days post-challenge) were also tested for HI antibodies to determine whether vaccination produced a “sterilizing” immunity that would prevent seroconversion in challenged ferrets. Ferrets in the mock and FluMist® vaccinated groups experienced a dramatic (>500-fold) increase in HI titer, indicating incomplete or no protection from challenge. In contrast, there was no significant increase in HI titer in the CodaVax-H1N1 group post-challenge (Table 1), suggesting that vaccination with a single dose of 5 x 10^3^ PFU CodaVax provided sterilizing immunity against homologous challenge.

## Discussion

The most effective countermeasures against infectious diseases are vaccines. The leading health security threat to the United States and the globe, as stated by the WHO and the CDC, is pandemic influenza. Current egg-reliant vaccine technologies cannot respond quickly to a pandemic, taking 6–9 months to generate a countermeasure. Furthermore, most new flu vaccine technologies are antigen-focused and require high antigen-per-dose, making them incapable of rapidly responding to an outbreak (cannot scale rapidly for mass use). Thus, there is a need for both a vaccine platform that can rapidly respond to pandemic AND a platform that yields an anti-pandemic vaccine that is scalable for mass distribution.

*SAVE*, a unique technology, relies on computer-aided design and chemical DNA synthesis to manufacture vaccine candidates rapidly, effectively, and safely. In this work, we produced a LAIV candidate, CodaVax-H1N1, that protects animals at an extremely low dose (5 x 10^0^ PFU) while being highly attenuated (LD_50_ >1 x 10^4^ PFU) in DBA/2 mice, yielding an unparalleled margin of safety. This vaccine was also successful in protecting ferrets at a dose of 5 x 10^3^ PFU, as measured by reduction of viral load in nasal washes, turbinates, and lungs post-challenge. Ferret morbidity post-challenge (weight loss and increased body temperature) were also prevented after vaccination with a low dose of CodaVax-H1N1.

In all except one ferret vaccinated with CodaVax-H1N1, post challenge (Day 49) HI titers to H1N1pdm influenza A virus did not increase but remained the same compared to Day 28 HI titers. The stable level of HI antibody indicated that these animals had experienced a sterilizing immune response after CodaVax-H1N1 vaccination at a low dose of 5 x10^3^ PFU. The high post-vaccination anti-HA antibodies (Day 28 HI titers of 640–1280) for the CodaVax-H1N1 vaccinated ferrets may have sequestered all viral particles, preventing or minimizing exposure to challenge virus. Conversely, ferrets in both FluMist® vaccinated groups responded with a boost in HI titer following challenge (Day 49), suggesting non-sterilizing immunity from the vaccine. This notion is supported by the clinical findings. At a high dose (1 x 10^7^), FluMist® provided some level of protection by reducing shedding by at least one day when compared to controls and protecting from a lung infection; however, significant weight loss was still observed, and virus was shed to high titers (comparable to controls) indicating this vaccination strategy would likely result in virus transmission. These ferret studies show the superiority of live-attenuated vaccination with CodaVax-H1N1 compared to FluMist®, the only licensed LAIV, which uses cold-adapted backbones from outdated influenza strains isolated in the 1960s.

At a 5,000-fold lower dose, CodaVax-H1N1 vaccination triggered much higher levels of HI antibodies and resulted in no detectable challenge virus in the nasal turbinates or lungs in ferrets vaccinated after a single dose of only 5 x 10^3^ PFU. FluMist® vaccinated ferrets showed reduced challenge virus load in the lungs, but high levels of virus were present in nasal turbinates and in nasal washes collected post-challenge.

In ferrets, a 5 x 10^3^ PFU dose of CodaVax-H1N1 vaccine protected so robustly that perhaps the vaccine dose can be reduced still further. Additional studies are also needed to assess the ability of *SAVE* attenuated influenza vaccines to elicit broadly protective and CTL mediated immunity. These findings support future research into the use of codon pair deoptimized vaccines for influenza and other viruses. As of the time of writing, CodaVax-H1N1 successfully completed a Phase I clinical trial and was demonstrated to be safe in humans. Clinical study results with CodaVax-H1N1 will be described in future publications.

## Materials and methods

### Cells and viruses

MDCK (Madin-Darby canine kidney), HEK-293T (Homo sapiens embryonic kidney expressing SV40 Large T antigen), and A549 (adenocarcinomic human alveolar basal epithelial) cells acquired from the American Type Tissue Culture Collection (ATCC, Manassas VA) were grown in Dulbecco’s modified Eagle’s medium (Gibco; MEM) with 10% fetal bovine serum (Hi-Clone; FBS).

A/CA/07/2009, CodaVax-H1N1, and mouse-adapted A/CA/07/2009 (ma) were constructed as reported previously with an 8-plasmid system [7] and transfection into HEK-293T/MDCK cell co-culture. The HA and NA gene segments of A/CA/07/2009 were redesigned using a computer algorithm and synthesized into a pUC57 plasmid backbone by Genscript. Mouse-adapted (CA07ma) virus was created by introducing two mutations into HA that had been previously observed in the closely related A/CA/04/2009 virus[21]. CodaVax-H1N1 was created by engineering the HA and NA genomic segments using our SAVE algorithm [7]. Viruses were generated using reverse genetics[8] and grown in MDCK cells using Infection Medium: MEM supplemented with Penicillin/Streptomycin (2x, Gibco), 2μg/mL TPCK-Trypsin (Sigma-Aldrich), and 0.2% bovine serum albumin (BSA; Sigma-Aldrich). FluMist® Quadrivalent (Catalog# 17695; Lot# CK2137; Expiration date: February 9, 2015) was purchased from Moore Medical (Farmington, CT) was stored at 4C and used prior to expiration.

### Vaccination of mice

DBA/2 mice used in these experiments were 34–37 days old, male, and ordered from Charles River Laboratories (Wilmington, MA). BALB/c mice were 5–6 weeks old, male, and ordered from Taconic (Hudson, NY). Mice were housed in standard conditions at the DLAR, Stony Brook University. Infected mice were monitored daily for morbidity (body weight) and mortality. Mortality was determined using humane early-endpoints including the presence of observed clinical signs (piloerection, hunched rodent posture) and weight loss ≥20%.

Groups of male DBA/2 mice or BALB/c mice (n = 5) were vaccinated intranasally with 25 μL CodaVax-H1N1 at different doses. On day 28 post-vaccination, sera were collected, and hemagglutination inhibition (HI) assays were performed with 0.5% Turkey red blood cells as described in the protocol of the World Health Organization’s Manual on Animal Influenza Diagnosis and Surveillance [22]. After serum collection, DBA/2 and BALB/c mice were challenged with 500 LD_50_ wild-type virus or mouse-adapted CA07 (10^5^ PFU), respectively. To lethally challenge BALB/c mice, we used mouse-adapted A/CA/07/2009 (CA07ma) which was generated by introducing two mouse adaptation mutations in the HA genomic segment. These adaptation mutations were previously described for the closely related H1N1pdm strain A/California/04/2009 [21]. A/California/04/2009 (“CA04”) and A/California/07/2009, which were isolated from a parent/child patient pair, differ only in a single amino acid within HA [23].

### Hemagglutination-inhibition assays of ferret sera

Serum samples taken from ferrets were treated with receptor destroying enzyme (RDE) (Denka Seiken, Tokyo, Japan) to eliminate inhibitors of nonspecific hemagglutination. RDE was reconstituted per the manufacturer’s instructions. Serum was diluted 1:3 in RDE and incubated 18–20 hours in a 37°C ± 2°C water bath. After the addition of an equal volume of 2.5% (v/v) sodium citrate, the samples were incubated in a 56 ± 2°C water bath for 30 ± 5 minutes. 0.85% NaCl was added to each sample to a final serum dilution of 1:10 after the RDE treatment. The diluted samples were then diluted into two-fold dilutions in phosphate buffered saline (PBS) then incubated with 4 hemagglutinating units of A/California/07/2009 (H1N1pdm). After incubation, 0.5% turkey red blood cells were added to each sample and incubated for 30 ± 5 minutes. Presence or absence of hemagglutination was then scored.

### Vaccination of ferrets

Thirty male ferrets (Triple F Farms, Sayre, PA) were approximately 5 months of age (152 days old) at the time of study initiation. The body weight ranges were 1.04–1.36 kg and were certified by the supplier to be healthy. Ferrets (n = 6) were inoculated intranasally with a single dose of 200 μl of 5 x 10^3^ PFU of CodaVax-H1N1 on day 0. Control groups were inoculated intranasally with 200 μl of 5 x 10^3^ PFU of FluMist® Quadrivalent, 200 μl of 1x10^7^ PFU of FluMist® Quadrivalent (a full commercial dose licensed for human use in 2014/2015), or mock vaccinated with 200 μl PBS (Gibco). Ferret body temperatures, weights, and clinical signs were monitored daily for 7 days post-inoculation. Blood was collected prior to inoculation (day -4) and days 28, and 49 and kept at ≤ -65°C until measurement of antibody titer by HI assay. All ferrets were challenged with a dose of 500 μl of 1x10^6^ TCID_50_ of wild-type A/California/07/2009 influenza virus on day 35; 5 weeks after the vaccines were administered. Ferret body weight, body temperature, clinical symptoms were monitored for 14 days after challenge and nasal washes and organs collected. Nasal washes were collected from challenged ferrets (3 per group) on days 1 through 5 post-challenge (days 36, 37, 38, 39, and 40) and the samples kept at ≤ -65°C for virus titration. On day 2 post-challenge (day 37), challenged ferrets (3 animals per group, total 12 animals) were euthanized and nasal turbinates as well as lungs were collected and were stored at ≤ -65°C until virus titration. Blood was collected 14 days post-challenge (day 49) and all surviving animals euthanized. The outline of the ferret experiments is detailed in Table 2.

**Table 2:** Outline of ferret experiments.

Group	Vaccine	N	Vaccination (Day)	Challenge (Day)	Nasal Washes (Days)	Organs^1^ (Days)	Serum Collections
1	CodaVax-H1N1	6	0	35	36–40	37	28, 49
2	FluMist®	6	0	35	36–40	37	28, 49
3	FluMist®	6	0	35	36–40	37	28, 49
4	Vehicle (Mock Control)	6	0	35	36–40	37	28, 49

^1^Lungs and nasal turbinates.

### Ethics statement

All mouse experiments conducted in this study were approved by the Stony Brook University Institutional Animal Care and Use Committee (IACUC) under protocol number 749202 using guidelines from *Guide for the Care and Use of Laboratory Animals*, 8^th^ Edition. Mouse experiments were conducted at the Office of Laboratory Animal Welfare accredited Division of Laboratory Animal Resources at Stony Brook University (OLAW Assurance #: D16-00006).

Ferret experiments were conducted at IIT Research Institute, Life Sciences Group, Chicago IL. IITRI animal laboratories are fully AAALAC accredited and are registered with the USDA; IITRI has an approved animal welfare assurance from NIH-OLAW (D16-00299). Euthanasia of ferrets was conducted using the recommendations of the American Veterinary Medical Association.

### Statistical analysis

Statistical analyses and figures were created using GraphPad Prism version 8.0.2 for Windows, GraphPad Software, La Jolla California USA, www.graphpad.com.
